# CALCB splice region pathogenic variants leading to plasma cell neurotropic enrichment in type 1 autoimmune pancreatitis

**DOI:** 10.1038/cddis.2017.32

**Published:** 2017-02-02

**Authors:** Qi-cai Liu, Falin Chen, Chao-yang Wu, Feng Gao, Ze-hao Zhuang, Jin-tong Chen, Bin Cai, Tianming Zhang, Ling Guo, Li-qing Lin, Cheng-fei Zhao, Xin-hua Lin

**Affiliations:** 1 Department of Laboratory Medicine, The 1st Affiliated Hospital, Fujian Medical University, Fuzhou, China; 2 Fujian Provincial Center for Clinical Laboratory, Fujian Provincial Hospital, Fuzhou, China; 3 Department of Orthopedics, The 1st Affiliated Hospital, Fujian Medical University, Fuzhou, China; 4 Department of Pathology, The 1st Affiliated Hospital, Fujian Medical University, Fuzhou, China; 5 Department of Gastroenterology, The 1st Affiliated Hospital, Fujian Medical University, Fuzhou, China; 6 Department of Neurology, the 1st Affiliated Hospital, Fujian Medical University, Fuzhou, China; 7 Department of Pharmaceutical Analysis, Fujian Medical University, Fuzhou, China

## Abstract

Recently, we have demonstrated that PRSS1 mutations cause ectopic trypsinogen activation and thereby result in type 1 autoimmune pancreatitis (AIP). However, the molecules involved in inducing obliterative vasculitis and perineural inflammation in the pancreas are not well-described. The present study applied whole-exome sequencing (WES) to determine the underlying etiology and revealed novel missense splice region variants, *CALCB* c.88T>C (p.Ser30Pro) and IR [1]-mutants, in 2 of the 3 families and 2 of 26 unrelated patients with type 1 AIP. *In vitro*, both of the mutants displayed decreased βCGRP, ERK1/2 phosphorylation, and co-localized with endoplasmic reticulum and Golgi apparatus. The novel pathogenic variant identified in this case should contribute to our understanding of the expanding spectrum of AIP.

Type 1 autoimmune pancreatitis (AIP) is the pancreatic manifestation of a systemic fibroinflammatory IgG4-related disease. It is often misdiagnosed as pancreatic cancer.^1, 2, 3^ Knowledge of genes' mutations may be valuable in making immediate informed treatment choices and further therapeutic discoveries.

During the last two decades, genetic factors have been identified in patients with chronic pancreatitis. Mutations in protease serine 1 (PRSS1) (OMIM 276000), cystic fibrosis transmembrane conductance regulator (CFTR) (OMIM 602421), and pancreatic secretory trypsin inhibitor (SPINK1) (OMIM 167790) were causally linked to the pathogenesis of chronic pancreatitis.^4, 5^ Recently, we have reported PRSS1_IVS 2+56_60 delCCCAG and PRSS1_p.Leu81Met cause ectopic trypsinogen activation resulting in AIP.^6, 7^ Moreover, we have observed the perineural inflammation and anti-collagen type IV antibodies co-localized with subepithelial IgG4 deposits along the capillary walls and surrounding nerve fibers in almost all patients,^8^ which highlights the involvement of neural factors in the formation of autoimmune pancreatitis.

Recent advances in understanding calcitonin gene-related peptide (CGRP) biology, central pain processes, and cerebellum biology now suggest that vascular activation may be just one of the several factors involved in migraine pathogenesis.^9, 10^ CGRP is a potent microvascular dilator neuropeptide, considered to play an essential role in neurogenic vasodilatation and maintaining functional integrity in peripheral tissues, and it was known to downregulate the immune response and influence the key processes in autoimmunity.^10, 11, 12, 13^ CGRP exists in two distinct isoforms: (1) αCGRP, which is the product of alternative splicing of the calcitonin gene (*CT/CALCA*) in neurons. (2) calcitonin gene-related peptide beta (*CALCB*), which has been discovered to form βCGRP, expressed primarily in the enteric sensory system, gut, and inner organs. In humans, the two isoforms, αCGRP and βCGRP, differ by three amino acids but have same biological effects in the vasculature. The primary neurons express more αCGRP than βCGRP, whereas enteric neurons almost exclusively express βCGRP.^14^

The best-known function of CGRP is its effect on the peripheral vasculature. It acts on the smooth muscle cells and causes vasodilatation via a non-endothelial mechanism through the activation of adenylate cyclase.^15^ The release of perivascular peptides relaxes cerebral arteries concomitant with the stimulation of cyclic AMP (cAMP) accumulation or release of an endothelium-derived relaxing factor. In the periphery, CGRP also has been known to modulate the neuromuscular junctions by inhibiting the expression of acetylcholinesterase,^16, 17^ which is involved in inflammation within the airways, gastric secretions, and intestinal mobility.^18^ It may dampen the immune response primarily by modifying antigen presentation in a variety of antigen-presenting cells and stimulating naive T cells in the primary immune response.^19^

Moreover, in the pancreas, trypsin activates the proteinase activated receptor-2 (PAR2) on sensory nerve endings evoking the local release of CGRP, which stimulates the extravasation of plasma proteins, infiltration of neutrophils, vasodilation (neurogenic inflammation),^20^ and joints.^21^ In the present study, we try to reveal the role of the CALCB mutations in development of obliterative vasculitis and perineural inflammation and the underlying mechanism(s).

## Results

### Clinical characteristics

All of the four patients (subjected to WES) were admitted with jaundice. Imaging analysis revealed pancreatic enlargement with extrahepatic biliary dilation. Laboratory examination showed increased serum IgG4 (10.22 g/l; range, 3.56–30.15 g/l), IgG (20.35 g/l; range, 15.59–47.12 g/l), IgE (552 IU/ml; range, 128–1450 IU/ml), total bilirubin (152.3 μmol/L; range, 75.6–389.4 μmol/l), and direct bilirubin (105.8 μmol/l; range, 45.8–288.6 μmol/l). All of the patients were treated with surgery.

### Gene mutations analysis

**Figure 1:**
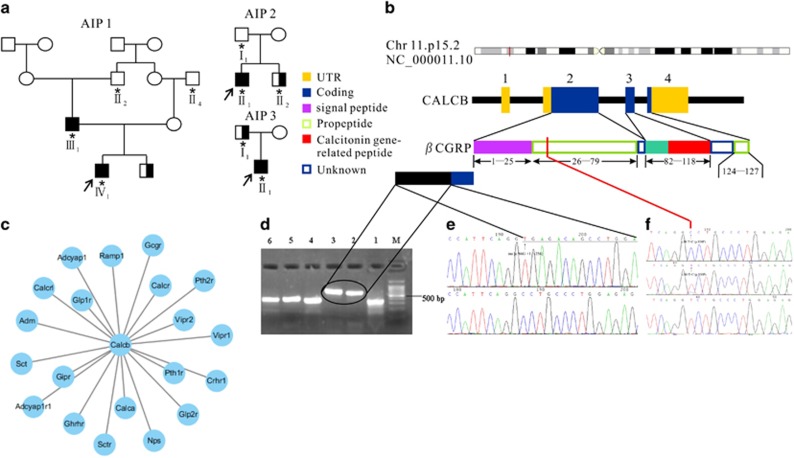
Identification of *CALCB* mutations in type 1 AIP. (**a**) The pedigrees of the three families affected by type 1 AIP (AIP1, AIP2, and AIP3), AIP patients (▪), Type II Diabetes Mellitus patients (▪) and their family normal members (○□), DNA collected (*), Proband (↗). (**b**) βCGRP protein domain structure. The *CALCB* gene contains five exons that encode several domains in the βCGRP protein, including two propeptide domains, two unknown domains and one signal peptide domain. (**c**) Protein–protein interaction network of CALCB. (**d**) RT-PCR analysis of *CALCB* mRNA in peripheral blood mononuclear cells. M: Marker, Lane 1: normal control, lane 2 and lane 3: INS [c.86 G +1:+256], lane 4–6: normal control. (**e**) INS [c.86 G +1:+256] mutation and the resultant mutant mRNA retains the first intron which were validated by Sanger sequencing. (f) c.88T>C in exon 2 (homozygous: top; heterozygous: middle; normal: bottom). The location of the mutation is indicated by the vertical arrows

After data filtering, we obtained variants in the AIP families (Supplementary Figure 1a, b and c). The table lists the number of variants (SNPs and Indels) detected in AIP patients (Supplementary Figure 1d and e). We also considered the splice region variants detected in *CALCB* as the disease-causing mutation in AIP1and AIP2 families (Figure 1b). The candidate pathogenic variants shared by the proband and his parents in AIP family were validated; these were potentially present in most of the affected males, or were absent in their male family members as controls and had not been previously reported or if it had a prevalence below 0.1% in the 1000 Genomes variant database. Gene Ontology (GO) annotations related to this gene include hormone activity and neuropeptide hormone activity.

The important paralog of this gene are *CALCA* and receptor genes, such as vasoactive intestinal peptide receptor (*VIPR*), type 1 parathyroid hormone receptor (*PTH1R*), glucagon-like peptide 1 receptor (*GLP1R*), and calcitonin (*CALCR*) (Figure 1c). There were two splice region variants found in the current study, one was INS [c.86 G +1: +256], identified in patient II-1 from AIP2 family, which resulted in the retention of the first intron (IR [1]) as assessed by RT-PCR (Figure 1d) and validated by Sanger sequencing (Figure 1e). Another novel missense pathogenic mutation, splice region variant, *CALCB* c.88T>C (p.Ser30Pro) is found in patient III–1 and IV–1 from the family of AIP1 (Figure 1a). This variation is similar to that reported in dbSNP (rs772389365), ENST00000324229.10:c.89C>T, ENSP00000346017.5:p.Ser30Phe. It revealed that parent III-1 was heterozygous for this variation, which was also confirmed by Sanger sequencing (Figure 1f). Furthermore, we also found *CALCB* c.88T>C mutation in two unrelated sporadic AIP patients, which confirm the correlation between *CALCB* mutation and AIP, although any *CALCB* mutations were not found in the AIP3 family. In addition, both of the two mutations were not found in 520 unrelated control participants and 20 cases of chronic pancreatitis.

### Histopathology and immunohistochemistry

**Figure 2:**
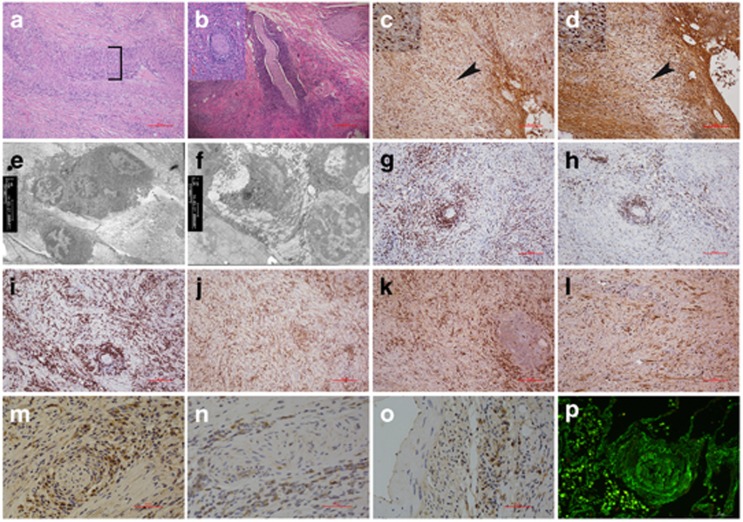
Histopathological and immunohistochemistry of pancreatic tissues from CALCB mutations (scale bar: 50 μm). (**a**) Typical features of pancreatic inflammation (fibrosis) and autoimmune disease (inflammatory infiltrates and vasculitis which depicted by bracket) (HE × 100). (**b**) Perineural inflammation: nerve fibers surrounded by an attenuated inflammatory infiltrate, concentric circle permutation (HE × 40) and nerve fiber crosses section (inset) (HE × 200). (**c**) Abundant IgG-positive plasma cells infiltration. (**d**) IgG4-positive lymphoplasmacytic cells. Enlarge as depicted by arrowheads and inset (× 200). (**e**) Electron microscopic examination indicates that dense lamina propria fibrosis and plasma cells and lymphocyte cells infiltration in the patient with the homozygous S30P *CALCB* mutation (× 3 800). (**f**) Collagen fiber diffuses hyperplasia, lymphocyte infiltration, and venous sinus occlusion (× 3 800). Immunohistochemical staining showed: CD3-, CD20-, CD138-, and CD68-positive infiltrating cells, which surrounded pancreatic nerve fibers. (**g**) CD3-positive T-lymphocytes are present within the lesion. (**h**) CD20 immunostaining demonstrates multiple B-cell aggregates. (**i**) CD138-positive are present around nerve fibers demonstrates that the infiltrating cells contain a large number of plasma cells. (**j**) CD68-positive is present macrophage. Polyclonality is established by the presence of both kappa (**k**) and lamda (**l**) light chain staining. βCGRP immunohistochemical analysis in pancreas from AIP: (**m** and **n**) βCGRP fails to stain nerve fibers from CALCB mutual pancreatic tissues, (**m** and **n**) βCGRP strong express in the inflammatory cells around the nerve fibers and (**o**) vasculature, (**p**) Immunofluorescence microscopy showed IgG-surrounded perivascular, resulting in obliterative vasculitis

H&E staining showed pancreatic inflammation (fibrosis), vasculitis (Figure 2a), and perineural inflammation (Figure 2b) from pancreatic tissue with CALCB p.Ser30Pro mutation. The sensory nerves and vasculature were surrounded by a severe lymphoplasmacytic infiltrate comprised predominantly of IgG-positive plasma cells (Figure 2c), and prominent IgG4-positive lymphoplasmacytic cells (Figure 2d). In addition, electron microscopy revealed inflammation within the lesion that contained lymphoplasmacytic infiltrate (Figure 2e) and associated fibrosis (Figure 2f). IHC showed CD3-, CD20-, CD138-, and CD68-positive infiltrating cells, which surrounded the pancreatic nerve fibers (Figures 2g and j), as well as the presence of both kappa- (Figure 2k) and lambda-positive cells (Figure 2l). Surprisingly, the βCGRP immunoreactivity was mainly found in the inflammatory cells surrounding the pancreatic nerve fibers (Figures 2m and n) and microvasculature (Figure 2o) but not in the neurons (Figures 2m and n) from the pancreatic tissue with *CALCB* mutations; immunofluorescence microscopy showed IgG-surrounded perivasculature (Figure 2p).

### Expression profiling of CALCB in p.S30P-transfected HEK293 cells by RNA-Seq

**Figure 3:**
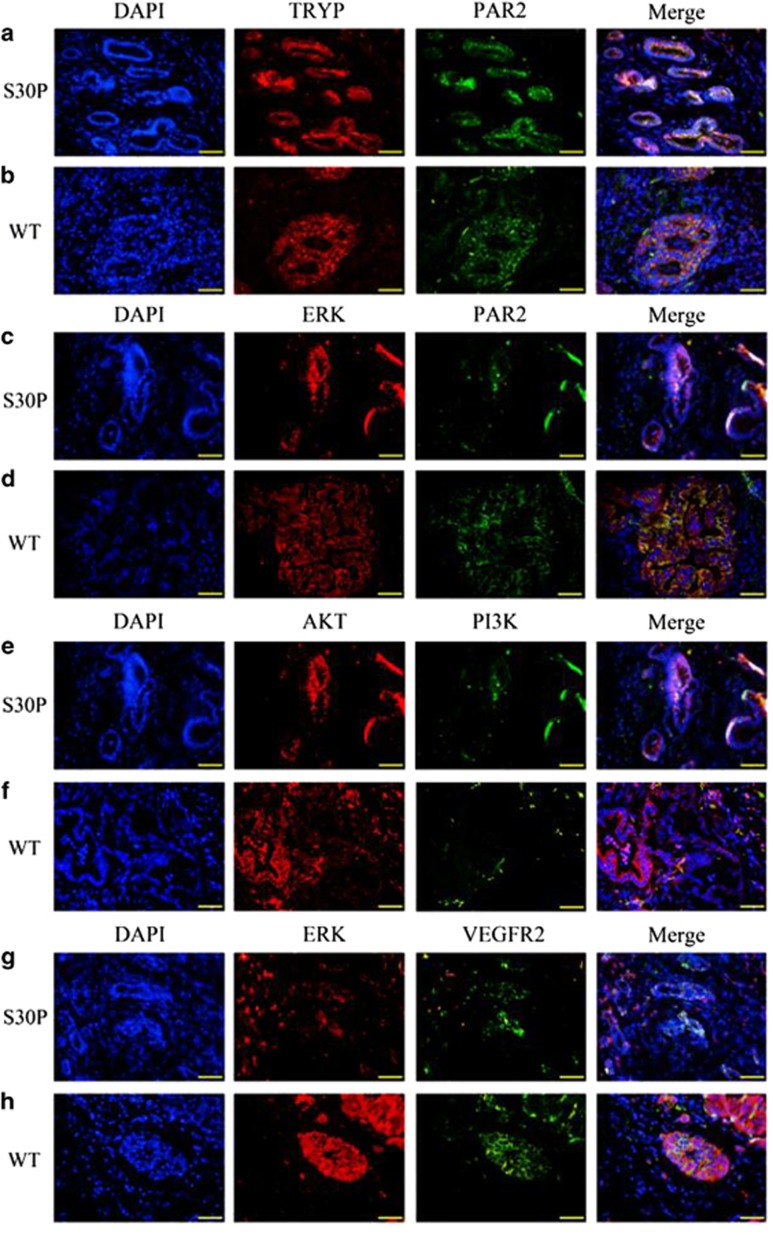
Representative immunofluorescence evidence for co-localization of PAR2/ERK/VEGFR2. Confocal immunofluorescence analysis showing diminutive areas of co-localized DNA in blue, TRYP in red and PAR2 in green, the overlap of TRYP and PAR2 (yellow staining in the merged image) indicates trypsin does co-expresses with PAR2 in the pancreatic tissues with CALCB_p.S30P mutations (**a**) and that of wild-type (**b**). Confocal immunofluorescence analysis showing small areas of co-localized DNA in blue, ERK in red and PAR2 in green, overlap of ERK and PAR2 (yellow staining in the merged image) in the endothelial and smooth muscle layers, indicates decreased ERK and PAR2 activity in CALCB_p.S30P mutations (**c**) than wild-type (**d**). Confocal immunofluorescence analysis showing small areas of co-localized DNA in blue, AKT in red and PI3K in green, in the endothelial and smooth muscle layers, indicates, double staining of AKT with PI3K showed that AKT co-expresses with PI3K in the tissues with CALCB mutation (**e**) and wild-type (**f**). Confocal immunofluorescence analysis showing diminutive areas of co-localized DNA in blue, ERK in red and VEGFR2 in green, indicates littler co-expresses of ERK and VEGFR2 in the tissues with CALCB_p.S30P mutation (**g**), while it does co-expresses in the wild-type (**h**). Original magnification: × 400. WT denotes wild-type

CALCB S30P mutation led to genes expression changes, which were associated with Glycolysis/Gluconeogenesis, Ubiquinone and other terpenoid-quinone biosynthesis, Morphine addiction, and Glycosphingolipid biosynthesis-globo series, activity up- or down-regulated, whereas most genes were involved in ‘Organismal Injury', ‘Gastrointestinal Disease', and ‘Hereditary Disorder'. The up-regulated genes were mainly observed in enriched Adipocytokine signaling pathway of the gene ontology in p. S30P-transfected HEK293 cells, and accordingly Ingenuity Pathway Analysis (IPA) predicted the top upstream regulator to be ‘Endocrine System Disorders' (*P*=9.67E-06, Activation z-score=2.526). The down-regulated genes are mainly enriched in the function of glycosphingolipid biosynthesis (*P*=9.26E-10, Activation *z*-score=−2.143). GOSim and SubpathwayMiner were employed for enrichment analyses of coding genes from each specific clusters based on GO terms and KEGG pathways, and each cluster was annotated with the enriched functions of the corresponding gene set, such as Glycolysis/Gluconeogenesis, Adipocytokine signaling pathway, and PPAR signaling pathway (Figure 3b) improved glycemic control, lipornetabolism, and insulin sensitivity.

### βCGRP expression in HEK293 cells

**Figure 4:**
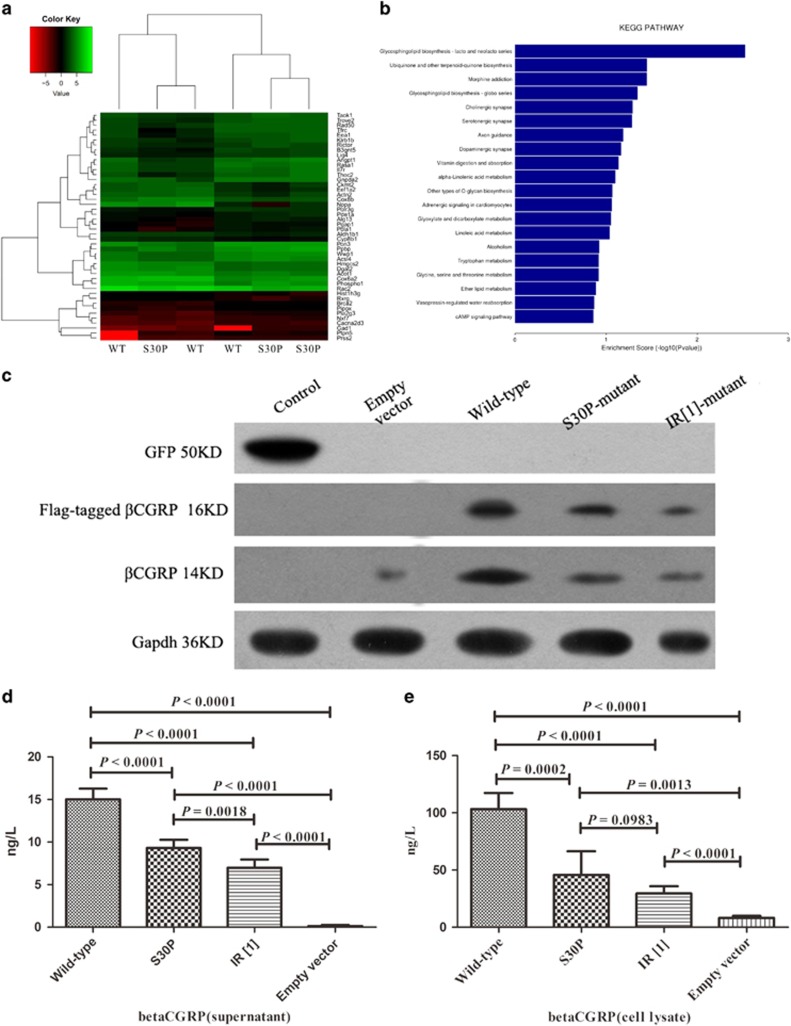
Identification of the differentially expressed genes of HEK 293 cells transfected with wild-type and mutants of *CALCB*. (**a**) Heat map demonstrating the most robust changes in the transcriptome of CALCB p.S30P and wild-type transfected HEK 293 cells (*n*=3). Color depth in the heatmap represents the different expression levels. Color key indicates metabolite expression value, blue: lowest; red: highest. (**b**) Importantly, KEGG pathway mapping of the entire set of differentially expressed genes revealed highly significant molecular interactions for KEGG. *X*-axis is an inverse indication of significance. (**c**) The expression of βCGRP in HEK 293 cells transfected with empty vector, wild-type *CALCB,* S30P, and IR [1]-mutants. Western blot was carried out with the use of antibodies against βCGRP or Flag. (**d**) The concentrations of βCGRP in the supernatant. (**e**) The concentrations of βCGRP in the cell lysate

**Figure 5:**
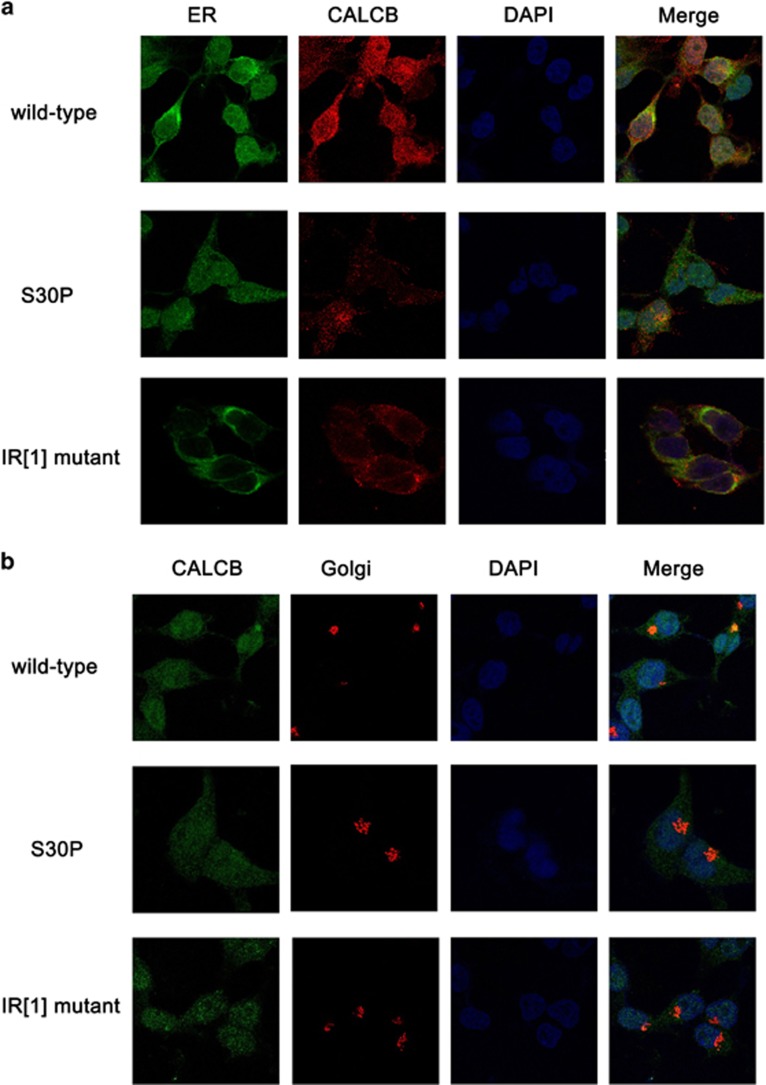
Representative images showing the localization of βCGRP with ER and Golgi in HEK293. Immunofluorescence staining of HEK293 cells with 4′, 6-diamidine-2-phenylidole dihydrochloride (DAPI), antibodies against Flag-tagged wild-type and mutant CALCB (red in (**a**)) and green in (**b**), ER (green in (**a**)), and Golgi (red in (**b**)). The yellow areas indicate the merging of CALCB and ER or Golgi straining in the podocytes

βCGRP in the supernatant and cell lysates from HEK293 cells transfected with empty vector-, wild-type CALCB-, p.S30P-, and p.IR [1] expression vectors was estimated by ELISA and/or Western blot. As shown in Figure 5c, βCGRP was significantly increased in p.S30P-, IR- [1], and wild-type CALCB-transfected HEK293 cells. The reason of beta-CGRP expression in empty vector may be that HEK293 cells themselves express a small amount of beta-CGRP (endogenous expression). The supernatant concentration of the βCGRP mutants was lower than that of wild-type (Figure 4d). Similarly, the cell lysate of mutant-types CALCB was significantly lower than that of the wild-type, however, there was no difference between the S30P- and IR [1]-type expression level (*P*=0.0983) (Figure 4e).

### Subcellular localization of βCGRP

Consistent with the wild-type recombinant CALCB, the fluorescence signal of the Flag-labeled mutant (S30P- and IR [1]-) βCGRP recombinant didnot coincided with the green fluorescent signals of the endoplasmic reticulum (ER) marker (Figure 5a) and the red signal of the Golgi apparatus marker (Figure 5b) in HEK293 cells, that is, only very few zones of yellow which would indicate co-localization with ER and Golgi apparatus in S30P and IR [1] mutants compared with wild-type. Thus, *in vitro,* the mutant βCGRP propeptides were fall to location with ER and Golgi.

### Located of Trypsin/PAR2/ERK/VEGFR2

In particular, the PAR2 receptor is activated by tryptase that cleaves the N-terminal domain of the receptor and signaling through the ERK/VEGFR2 pathway. To examine this possibility, we initially performed immunohistochemical (IHC) studies to determine whether CALCB*_*p.S30P mutations affect the co-localization of trypsin and PAR2 in the inflamed pancreas of IgG4-related AIP patients. We found that trypsin does co-expresses with PAR2 in the pancreatic tissues neither from patients with CALCB_p.S30P mutations (Figure 5a) and that of wild-type (Figure 5b). The cellular localization of the PAR2/ERK signaling pathway showed a rare overlap of ERK and PAR2 (yellow staining in the merged image) (Figures 5c and d) in the mutant-type (Figure 5c) than that of wild-type (Figure 5d) indicating that CALCB*_*p.S30P may affect the localization of ERK and PAR-2. Double staining of AKT with PI3K showed that AKT co-expresses with PI3K in the tissues with CALCB mutation (Figure 5e) and that of wild-type (Figure 5f). Intriguingly, ERK and VEGFR2 slightly co-localized in the tissues with CALCB*_*p.S30P mutation (Figure 5g) than that in wild-type (Figure 5h).

### βCGRP activates the phosphorylation of ERK1/2 resulting in autoimmune inflammation

**Figure 6:**
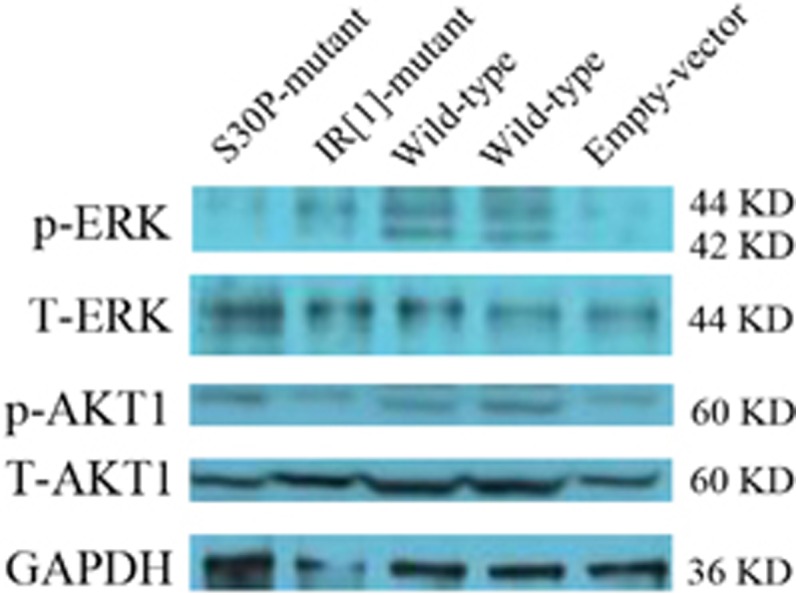
CALCB mutation effect the phosphorylation of ERK1/2. Phosphorylation of AKT1 on serine 473 (pAKT1, S473), total AKT1 protein. Phosphorylation of ERK1/2 (phospho-T202/204) and total ERK1/2 protein. GAPDH levels were assessed as a control for loading

To examine whether CALCB mutations effect on βCGRP activates the phosphorylation of ERK1/2, we performed a Western blot on the cell lysates from HEK293 cells transfected with empty vector, wild-type CALCB, p.S30P, and p.IR CALCB mutations, which indicated that p.S30P and p.IR CALCB mutations induced a decrease in the phosphorylation of ERK1/2 (Figure 6).

**Figure 7:**
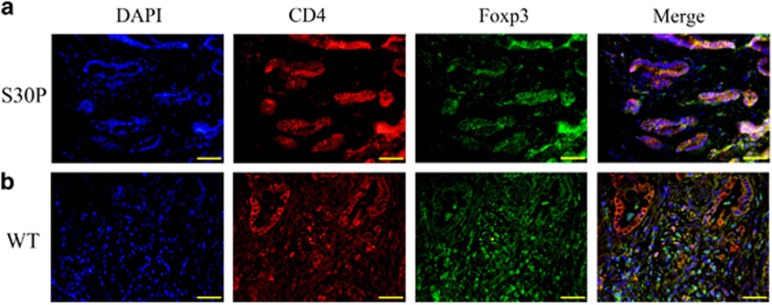
CALCB mutation effect the T_reg_ cells dispense. Co-localization of DNA (blue), CD4 (red), and Foxp3 (green) indicates T_reg_ cells formation. Original magnification: × 400. WT denotes wild-type

In this study, we also found that the distribution of CD4^+^Foxp3^+^ (T_reg_) cells was significantly different in pancreatic tissues from CALCB*_*p.S30P mutations and wild-type. A majority of the T_reg_ cells were distributed around pancreatic ductal or blood vessels in the tissues with CALCB mutations (Figure 7a), while they were scattered in wild-type from chronic pancreatitis (Figure 7b).

## Discussion

Growing evidence suggests that CGRP prevents excessive immune activation, inhibiting proinflammatory cytokine injury and maintaining a balance between pro- and anti-inflammation without the pancreas.^22^ Moreover, CGRP reduced the leukocyte infiltration into the pancreatic tissue and increased the pancreatic blood flow, permitting removal of active digestive enzymes and mediators of inflammation, thereby attenuating the pancreatic damage in pancreatitis.^10, 23^ The present study found that the *CALCB* splice region variants (*CALCB_*p.S30P and IR [1]) resulted in a decreased βCGRP. This revealed a new meningeal neurovascular mechanism by showing that trypsin stimulation of PAR-2 and βCGRP induces a marked vasodilatory response in the obliterative vasculitis and perineural inflammation in pancreatic lesions of patients with IgG4-related AIP.

βCGRP is synthesized by dorsal root ganglia, transported to the nerve endings along the axon, and stored in vesicles. Immunocytes also synthesize a small amount of βCGRP after prolonged stimulation and induction.^23, 24^ Therefore, lymphocyte aggregation may compensate for the decreased or absent expression of neurogenic βCGRP caused by CALCB mutations, which may explicate that the pancreatic nerve fibers in patients with AIP were frequently encompassed by immune cells. Moreover, CGRP-containing fibers are closely associated with blood vessels, where CGRP release is localized perfectly for its role as a vasoactive mediator, resulting in the formation of typical pathological characteristics related to neural immunity, such as neuritis and vasculitis.

To study the intracellular transportation of βCGRP, we used the expression vectors in which both *CALCB* and *EGFP* have independent promoters. In this construct, βCGRP was Flag-tagged, while the expression of EGFP was an indicator of the efficiency of transfection. The HEK293 cells transfected with *CALCB* 30 Phe and IR[1]-mutants exhibited ~50 and 33% βCGRP concentration of that observed in cells transfected with wild-type *CALCB*, in the cell supernatants or lysates. In addition, the mutant βCGRP propeptides, *in vitro*, were fall to location with Golgi and ER. Thus, we speculated that *CALCB* mutations might affect βCGRP expression.

Moreover, PAR2 regulates the gastrointestinal motility and secretion and may play a vital role in autoimmune disease.^20, 25^ The trypsin released during inflammation and injury cleave and activate PAR2 on the terminals of nociceptive spinal afferent neurons and directly act on these receptors in eliciting inflammation and pain by means of neurogenic pathways.^26, 27^ The release of these inflammatory mediators are consequences of the complex interplay of PI3K/ERK1/2 kinase.^28^ Therefore, we explored the possible involvement of *CALCB* mutations in the trypsin-induced AIP. Our results indicated that trypsin co-expressed with PAR2 in the pancreatic tissues neither from the patients with *CALCB* mutations nor that of the wild-type, it maybe supporting the effects of trypsin in the AIP.^6, 7^ These findings are in agreement with previous data demonstrating that ectopic trypsinogen activation can directly stimulate the inflammation in the pancreas.^6, 7^ Moreover, trypsin and tryptase acting on PAR-2 receptors, stimulate the release of neuropeptides from slices of the spinal cord (Supplementary Fig. 2). The present study also showed that *CALCB* mutations maybe reduce the phosphorylation level of ERK and affect the co-localization of ERK and VEGFR2.

Collectively, the gene expression profile was confined mainly to endocrine and neural genes, which is consistent with the current views on the immunopathogenesis of AIP. These features, in addition to perivascular inflammatory cell infiltration, suggest that vasculitis in AIP was the cause of the neuropathy.

## Materials and Methods

### Study population

Three unrelated families of Chinese origin were included in the study (Figure 1). Patients IV-1 and III-1 from AIP1, patient II-1 from AIP2, and patient II-1 from AIP3 fulfilled the diagnostic criteria^19, 20^ on the basis of the histological examination of pancreatic specimens obtained during surgery. Moreover, 26 unrelated patients with type 1 AIP, 20 patients with chronic pancreatitis, and 520 unrelated healthy volunteers, were included as a control (Supplementary Figure 3).

### Whole-exome sequencing (WES)

WES was performed on DNA extracted from peripheral blood obtained from patients IV-1 and III-1 of AIP1 family, patient II-1 of AIP2 family, and patient II-1 of AIP3 family and their male family members (II-2 and II-4 from AIP1, I-1 and II-2 from AIP2, and I-1 from AIP3) shown in Figure 1a. The samples were sequenced on an Illumina HiSeq2500 platform (100 bp X2) after exome capture with SureSelectXT Human All Exon V5 kit (Agilent Technologies, Santa Clara, CA, USA). After raw data processing, reads were mapped to human genome reference hg19 using BWA (bio-bwa.sourceforge.net) with realignment around the known Indels from the 1000 Genomes Pilot study (http://www.1000genomes.org/category/pilot-study). The recalibration of the base quality scores was performed according to the recommendations of the GATK Best Practices (http://www.broadinstitute.org/gatk). Variant calling and independent filtering were performed using GATK Unified Genotyper and SAMtools mpileup.

Only variants with a mean read depth of at least × 10 and a minimum quality score of 30 were considered for further analyses. This low coverage threshold, even though it may include a certain number of false positives, was chosen to consider the largest number of possible variants. The Phred-scaled quality score of 30 corresponds to a base calling accuracy of 99.9%.

As AIP is rare in the general population, variants with a minor allele frequency >1% in 1000 Genomes (October 2014 release, all subjects) and NHLBI-ESP 6500 Exomes (all subjects) databases were excluded.

An autosomal recessive inheritance model was used for the identification of candidate genes in families. Therefore, we analyzed the exome data considering homozygous variants shared by the affected for which the parents resulted heterozygous. The candidate pathogenic variants were validated by Sanger sequencing.

### Immunohistochemistry and microscopy

Pancreatic tissues from patients with AIP were used for hematoxylin and eosin (H&E) staining and immunohistochemistry (IHC) analysis. IgG, IgG4, CD3, CD20, CD138, κ, λ, and βCGRP antibodies were obtained from Santa Cruz Biotechnology (Santa Cruz, CA, USA). To identify the ultrastructural characteristics of AIP, pancreatic tissues were fixed with glutaraldehyde (2% v/v) in 0.1M phosphate buffer (pH 7.4), dehydrated through a gradient of ethanol, and embedded in EPON epoxy resin. Ultrathin sections of 90-nm thickness were sliced from each block, and double-strained with uranyl acetate and lead citrate and observed using transmission electron microscopy. Deposits of IgG4 were detected by direct immunofluorescence microscopy on frozen sections using 100-fold diluted fluorescein isothiocyanate-labeled antibodies specific to mouse or rabbit IgG (Dako, Hamburg, Germany).

### Transfection of HEK293 cells *in vitro*

The GV287 plasmid was linearized with AgeI to obtain a *CALCB* fragment of 384 bp (c.88T>C mutant or wild-type) or 640 bp (INS [c.86 G +1: +256]). The fragment was sub-cloned into pcDNA3·1(−) (Invitrogen, Carlsbad, CA, USA). After transfection of *CALCB* expression vectors or the empty control in HEK293 cells for 24 h, the cells were harvested, and total protein was extracted for Western blot and enzyme-linked immunosorbent analyses (ELISA).

### RNA-SEQ

Transcriptome deep sequencing (RNA-seq) was performed using total RNA isolated from wild-type-, and S30P mutant-transfected cells. Three individuals from each genotypic group were randomly selected. Total RNA was extracted from frozen tissue using the SV Total RNA Isolation System (Promega, Madison, WI, USA) according to the manufacturer's instructions. The samples were sent to DRIGEN Co., Ltd. for RNA-seq library preparation using the TruSeq SBS Kit (75 cycles) and single-end sequencing on Illumina NextSeq 500 (Illumina, San Diego, CA, USA). RNA-seq reads were quality filtered using SolexaQA packages with default parameters, as well as, for the requisite length >70 bp for both ends of each read pair. The sequencing data have been submitted to the NCBI Sequence Read Archive. Genes that showed a significant (*P*<0.05) difference in transcript levels were termed as differentially expressed (DE) genes.

### Analysis of CALCB expression and ERK phosphorylation

To determine the expression of exogenous βCGRP among the empty vector-, wild-type-, S30P mutant-, and IR [1] mutant-transfected HEK293 cells, Western blot analysis was carried out using antibodies specific for Flag and βCGRP. GAPDH was used to assess the loading variation and enhanced green fluorescent protein (eGFP) for transfection efficiency and extracellular signal-related kinase 1/2 (ERK1/2) phosphorylation (phospho-T202/204). βCGRP levels in the supernatant and cell lysate were measured by ELISA (R&D Systems, Minneapolis, MN, USA).

Proteins from transfected HEK293 cells lysate were separated on 4–12% Tris-glycine gels and transferred to nitrocellulose membranes. Membranes were probed with antibodies directed against (phospho-T202/204) ERK1/2 (AP0472), total ERK (ABclonal, Wuhan, China, A1796), AKT1 (ABclonal, AP0004), (phospho-S473) AKT1 (ABclonal, AP0140), and GAPDH (ABclonal, AP5809).

### Subcellular localization of βCGRP

Ubi-MCS-3FLAG-SV40-EGFP was expressed in HEK293 cells, and immunofluorescence confocal microscopy was employed to determine its subcellular localization using GM130 antibody (BD Bioscience, San Jose, CA, USA) and anti-mouse-cy3 (Jackson ImmunoResearch, West Grove, PA, USA) for the Golgi apparatus, Flag antibody (Sigma, CA, USA) and anti-rabbit-647 (Jackson ImmunoResearch) for βCGRP, and calnexin antibody (Sigma) and anti-rabbit-647 for the ER.

### Localization of trypsin/PAR2, PAR2/ERK, PI3K/AKT, and CD4/FOXP3

PAR2 is reported to coexpress with CGRP by a subpopulation of primary spinal afferent neurons that control neurogenic inflammation.^29, 30^ Moreover, trypsin activation of PAR2 induces activation of ERK1/2 in order to regulate a variety of nuclear transcription factors by phosphorylation, which contributes to the effect of trypsin on gastrointestinal smooth muscle contraction.^31^ In the current study, confocal immunofluorescence microscopy was undertaken to determine the correlation of trypsin (TRYP) and PAR2, PAR2 and ERK, PI3K and AKT, and ERK and VEGFR2. Trypsin was detected with a rabbit anti-human antibody (ABclonal) and labeled with a goat anti-rabbit secondary antibody conjugated to Cy3. PAR2 was detected with a rabbit anti-human antibody (Wanlei, Shenyang, China) and labeled with a goat anti-rabbit secondary antibody conjugated to FITC. ERK was detected with rabbit anti-human antibody (Sangon, Shanghai, China) and labeled with a goat anti-rabbit secondary antibody conjugated to Cy3. AKT was detected with a mouse anti-human antibody (Sanying, Wuhan, China) and labeled with a goat anti-mouse secondary antibody conjugated to Cy3. PI3K was detected with a rabbit anti-human antibody (Wanlei) and labeled with a goat anti-rabbit secondary antibody conjugated to FITC. VEGFR2 was detected with rabbit anti-human antibody (Sangon) and labeled with a goat anti-rabbit secondary antibody conjugated to FITC. Nuclei were counterstained using 4', 6-diamidine-2-phenylidole dihydrochloride (DAPI).

Tregs were identified by staining with anti-CD4 and anti-Foxp3. CD4 was detected with a rabbit anti-human antibody (Sanying) and labeled with a goat anti-rabbit secondary antibody conjugated to Cy3. Foxp3 was detected with a mouse anti-human antibody (Santa Cruz) and labeled with a goat anti-mouse secondary antibody conjugated to FITC. Nuclei were co-stained using DAPI.

### Statistical analysis

All data were analyzed with SPSS version 18.0 statistical software (SPSS Inc., Chicago, IL, USA). Normal variation was expressed as mean±s.d. and analyzed by one-way analysis of variance followed by the least significant difference *t*-test among groups.
