# Supplementary figures and images for: CALCB splice region pathogenic variants leading to plasma cell neurotropic enrichment in type 1 autoimmune pancreatitis
Source: Cell Death Dis. 2017 Feb 2;8(2):e2591–. doi: 10.1038/cddis.2017.32 (PMC5386480; doi:10.1038/cddis.2017.32)

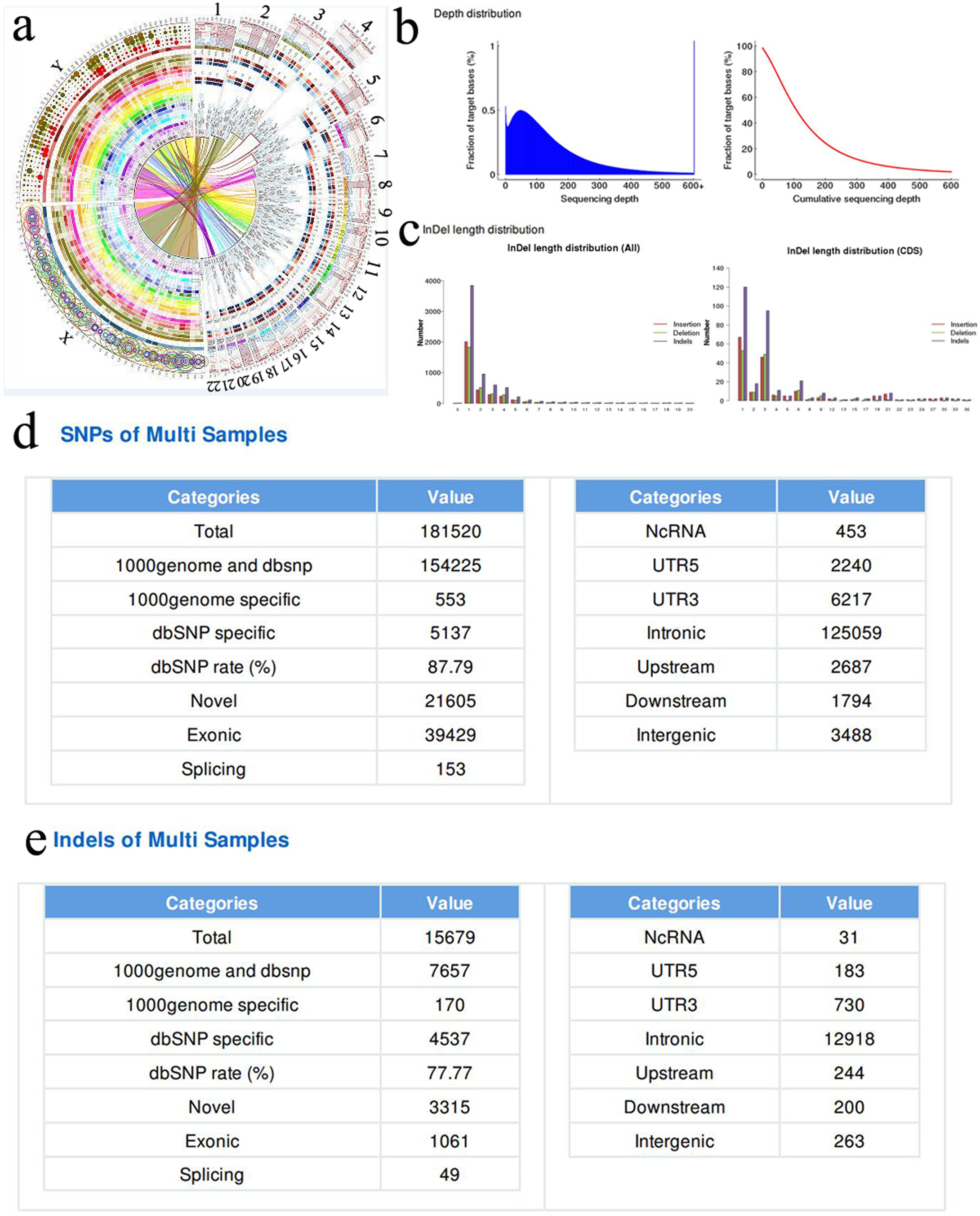

Supplement: Supplementary Information [file cddis201732x1.tif]

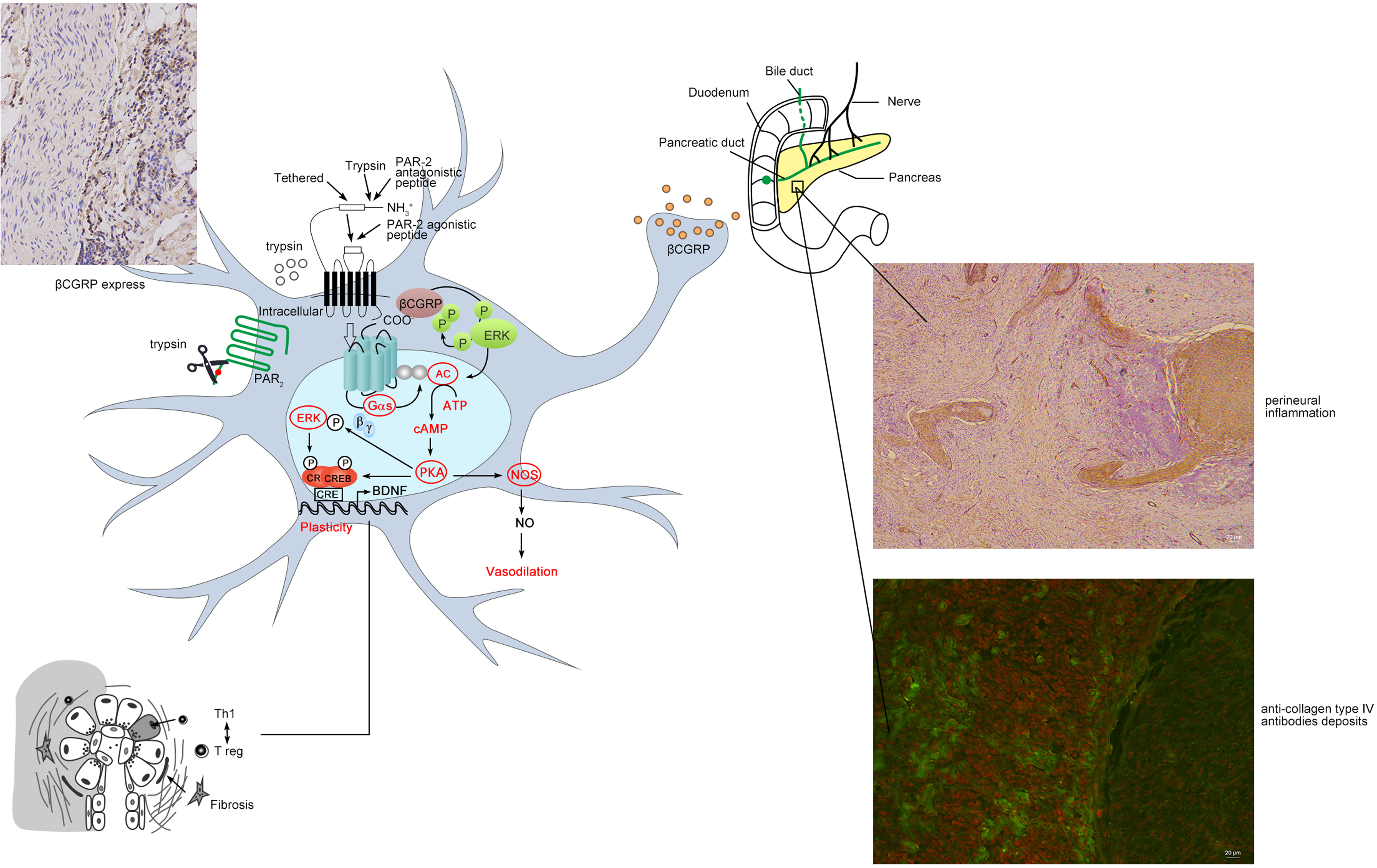

Supplement: Supplementary Information [file cddis201732x2.tif]

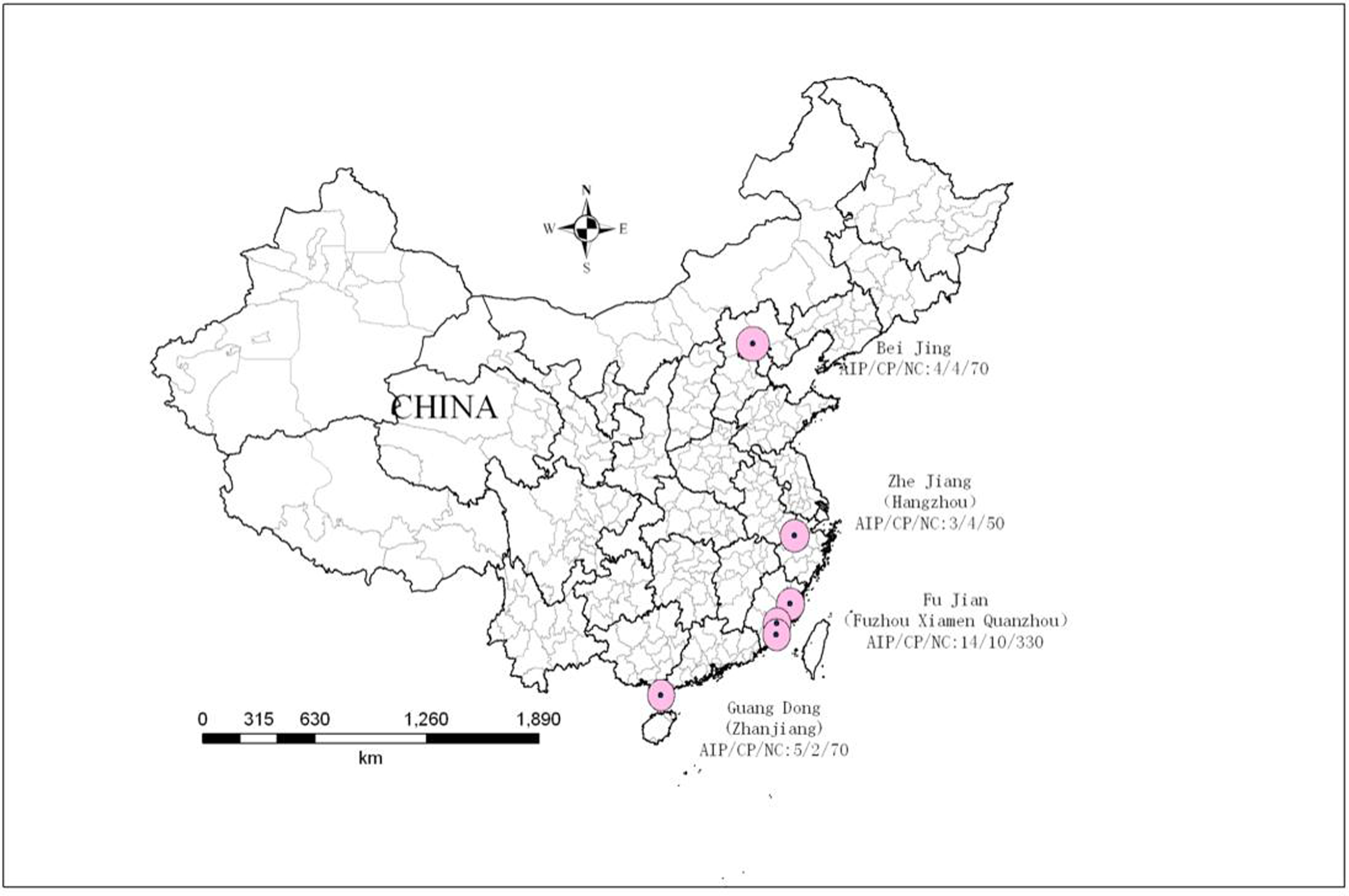

Supplement: Supplementary Information [file cddis201732x3.tif]
